# Efficacy of Credelio Quattro™ chewable tablets containing lotilaner, moxidectin, pyrantel, and praziquantel against *Ctenocephalides felis* and *Rhipicephalus sanguineus* infestations on dogs

**DOI:** 10.1186/s13071-026-07405-1

**Published:** 2026-04-27

**Authors:** Lisa Young, Xinshuo Wang, Scott Wiseman, William R. Everett, Riaan Maree, Utami R. DiCosty, Carin Rautenbach, Molly D. Savadelis

**Affiliations:** 1 Elanco Animal Health, Indianapolis, IN 46222 USA; 2 Elanco Animal Health, Bartley Way, Hook, RG27 9XA UK; 3 Bertek Inc., Greenbrier, AR 72058 USA; 4 Clinvet US, Waverly, NY 14892 USA; 5 TRS Labs Inc., Athens, GA 30607 USA; 6 Clinvet SA, Bloemfontein, South Africa

**Keywords:** Credelio Quattro, Credelio, Lotilaner, *Rhipicephalus sanguineus*, *Ctenocephalides felis*, Ectoparasitic infestations

## Abstract

**Background:**

Fleas and ticks can be found globally and are of both veterinary and human health concern due to their ability to transmit various vector-borne diseases. Heavy flea and tick infestations can result in significant blood loss, while flea infestations can result in intense pruritus. The use of safe and effective ectoparasiticides in veterinary medicine is a crucial part of protecting both pets and humans from infestations and transmission of vector-borne diseases.

**Methods:**

The efficacy of a novel endectocide, Credelio Quattro, containing lotilaner, moxidectin, praziquantel, and pyrantel, was evaluated in four masked studies: one against *Ctenocephalides felis* and three against the dose-limiting tick *Rhipicephalus sanguineus*. Dogs were orally administered placebo, Credelio Quattro™, lotilaner only (Credelio™, Study 2), or pyrantel only (Study 2) in a fed state on Day 0. Experimental infestations with *C. felis* were conducted on Days −1, 6, 13, 20, 29, and 35 with 100 adult fleas. Fleas were removed and categorized as either live or dead 24 h post-treatment and 24 h post-infestation thereafter. Experimental infestations with *R. sanguineus* were conducted on Days −2, 5, 12, 19, and 30 with 50 adult ticks. Ticks were removed and categorized as attached or unattached and then live or dead 48 h post-treatment and 48 h post-infestation thereafter.

**Results:**

Credelio Quattro demonstrated 100% (*P* < 0.0001) efficacy against *C. felis*, with no live fleas recovered at time points evaluated through 36 days post-treatment. Additionally, Credelio Quattro demonstrated 97.1–100% (*P* < 0.0001) efficacy through Day 30 against the established dose-limiting tick species, *R. sanguineus*. In Study 2, pyrantel demonstrated ≤ 46.6% (*P* ≥ 0.0601) efficacy while lotilaner provided ≥ 99.6% (*P* ≤ 0.0007) efficacy, confirming lotilaner as the acaricidal active ingredient in Credelio Quattro. Adverse events were reported in both treatment groups, including dermatitis, alopecia, lameness, and a chest wound. One dog in a Credelio Quattro treatment group experienced vomiting and bloody diarrhea that was considered possibly treatment-related.

**Conclusions:**

These studies confirm the safety and effectiveness of a single dose of Credelio Quattro for the treatment and control of *C. felis* and *R. sanguineus* infestations on dogs for 1 month.

**Graphical Abstract:**

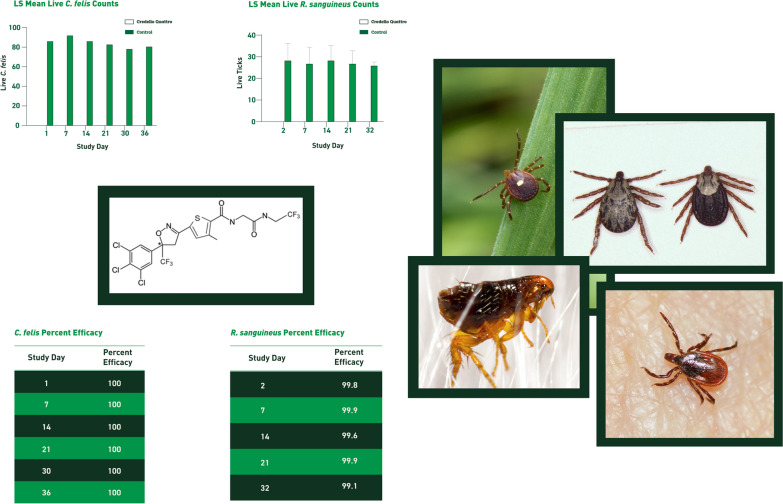

**Supplementary Information:**

The online version contains supplementary material available at 10.1186/s13071-026-07405-1.

## Background

Ectoparasites such as fleas and ticks are found globally, infesting a wide range of hosts including rodents, birds, companion animals, livestock, and humans. The most widespread ectoparasites globally include the cat flea, *Ctenocephalides felis*, and the brown dog tick, *Rhipicephalus sanguineus* [1]. The ixodid tick *R. sanguineus* thrives in warmer climates and is prevalent in North America, South America, Africa, Europe and Asia, but is able to survive in colder climates due to this tick’s adaptation to living indoors as well [1, 2]. As a four-stage life cycle tick, *R. sanguineus* eggs hatch into larvae, and then develop into nymphs and subsequently sexually dimorphic adults, with each life stage requiring a blood meal to progress. While *R. sanguineus* exhibits a strong host preference for canines, this tick species can also be found to infest felines and humans [3, 4]. Due to this host preference, dogs can be infested with large numbers of *R. sanguineus* at one time, potentially leading to anemia due to blood loss, inflammation at the bite locations, and development of secondary infections [2]. Several vector borne diseases can be transmitted by *R. sanguineus* including *Ehrlichia canis*, *Rickettsia rickettsii*, *Rickettsia conorii*, *Babesia vogeli*, and *Hepatozoon canis*, with the latter being transmitted through the ingestion of infected ticks and the former pathogens being transmitted through tick saliva during blood-feeding [1, 5, 6].

*Ctenocephalides felis*, from the family Pulicidae, is prevalent globally, infesting dogs, cats, and wildlife and even affecting humans [7–9]. Within 24–48 h after blood-feeding on a host, adult female fleas lay eggs that hatch within 1–10 days in the environment to the larval stage [7]. These free-living larvae feed on various organic materials and adult flea feces, completing two molts over approximately 5–11 days before developing into the pupal stage [7, 10]. Flea pupae produce a silk-like cocoon covered with debris in the environment and begin emerging as adults within 5–9 days after environmental stimulation. If no environmental stimulation such as heat, carbon dioxide, or physical pressure occurs, the pre-emerged adult can remain viable in the cocoon for months [11]. Adult fleas are capable of transmitting bacterial pathogens such as *Rickettsia typhi*, *Rickettsia felis*, and *Bartonella henselae* and are implicated in the transmission of *Mycoplasma haemofelis* [12, 13]. In addition to bacterial pathogen transmission, *C. felis* adults are the intermediate host for *Acanthocheilonema reconditum* and *Dipylidium caninum* [14, 15].

Effective control strategies to protect against ectoparasite infestations and transmission of vector-borne diseases include multimodal approaches such as elimination of suitable habitats and wildlife access in backyards, performing tick checks immediately after outdoor exposure, and use of effective ectoparasiticide products [7]. These strategies aim to greatly reduce overall exposure to ectoparasites and quickly kill ectoparasites before potential disease transmission can occur. The most recently developed drug class of ectoparasiticides in veterinary medicine are the isoxazolines, such as lotilaner, sarolaner, afoxolaner, and fluralaner. Isoxazolines are potent antagonists of insect GABA-gated chloride ion channels, providing robust efficacy against various ectoparasites such as ticks, fleas, mites, mosquitoes, and lice [16–20]. To provide the most complete protection, the Companion Animal Parasite Council (CAPC) recommends the use of broad-spectrum parasite control year-round against heartworm, gastrointestinal parasites, ticks, and fleas [21]. Additionally, regular year-round prophylaxis using approved products against fleas and ticks is recommended by the European Scientific Counsel Companion Animal Parasites (ESCCAP) to provide constant protection in areas with ongoing reinfestation risk and for owners traveling with pets [22].

Ectoparasites and endoparasites such as gastrointestinal nematodes and cestodes as well as vascular nematodes pose a relevant and simultaneous risk to dogs. Therefore, Credelio Quattro containing lotilaner, moxidectin, praziquantel, and pyrantel was developed as a combination product to broaden the activity spectrum of the monoproduct Credelio (lotilaner, Elanco Animal Health, Indianapolis, IN, USA) by including indications against endoparasites. The Credelio Quattro combination provides a safe and convenient option for veterinarians and pet owners to follow parasite control recommendations for continuous prophylaxis.

Routine administration of combination products has a significant positive impact on reducing the overall parasite burden exposed to both pets and people [23]. A comprehensive parasite control strategy breaks the life cycles of various parasites, preventing the establishment of ectoparasite (e.g., flea) infestations in the home [23] and limiting contamination of soil and water with the eggs of zoonotic intestinal worms [24, 25]. Furthermore, by maintaining the health of individual animals, this approach can reduce the reservoir of pathogens for vector-borne diseases, ultimately minimizing parasite-associated risks for pets and people [21].

During the development of lotilaner orally as the monoproduct Credelio for dogs, *R. sanguineus* was established as the dose-limiting tick species as compared to *Ixodes scapularis*, *Ixodes ricinus*, *Dermacentor variabilis*, *Dermacentor reticulatus*, and *Amblyomma americanum* [26, 27]. Therefore, as the least susceptible tick species for lotilaner, demonstration of efficacy against *R. sanguineus* provides substantial evidence for a product’s ability to achieve efficacy for *I. scapularis*, *D. variabilis*, and *A. americanum* as well. The objective of the studies described below was to evaluate the efficacy of a novel isoxazoline combination chewable tablet, Credelio Quattro, containing lotilaner, moxidectin, praziquantel, and pyrantel, for the treatment and control of *C. felis* and *R. sanguineus* infestations on dogs.

## Methods

Three studies (two in the USA and one in Europe) were conducted to evaluate the efficacy of Credelio Quattro against experimental infestations of *R. sanguineus* and one study against *C. felis*. All studies were masked and were conducted according to the World Association for the Advancement of Veterinary Parasitology (WAAVP) guideline for evaluating the efficacy of parasiticides for the treatment, prevention, and control of flea and tick infestations on dogs and cats [28] and VICH GL9 Good Clinical Practice guideline. The protocols were reviewed and approved by the study site’s animal care and use committee or the Elanco Animal Health Care and Use Committee prior to initiation.

### Animals

Purpose-bred male and female beagles or mongrel dogs, 10 dogs per treatment group, ranging from 10 months to 7 years and 9 months of age and weighing between 5.5 and 21.2 kg, were enrolled in each study. To be eligible for inclusion, dogs were required to be healthy, not pregnant or lactating, nor intended for breeding during the study and have pre-allocation infestation rates of ≥ 25% live fleas or live attached ticks. Throughout the studies, age-appropriate, commercially available diets and access to potable water ad libitum were provided. Study dogs were individually housed and provided appropriate enrichment items throughout the entire in-life phase. Housing was environmentally controlled, and a photoperiod of approximately 12 h light and 12 h darkness was maintained.

### Randomization and treatment

All studies utilized a randomized complete block design. Pre-treatment infestation live flea counts or live attached tick counts were used as the blocking factor, rank ordering dogs by counts from highest to lowest. In the event that two or more dogs had the same baseline flea or tick counts, dogs were ranked by their identification code in a descending manner. Within each block, assignment to treatment groups was completely random.

An outline of the treatment groups, species evaluated, and isolate origin for each of the studies is provided in Table 1. Each study evaluated the efficacy of Credelio Quattro as compared to a non-treated control group. Study 2 also evaluated the efficacy of lotilaner and pyrantel only as compared to the non-treated control. Inclusion of the lotilaner and pyrantel-only treated groups was performed to demonstrate that lotilaner contained in Credelio Quattro delivers the ectoparasite efficacy in this combination tablet.

**Table 1:** Summary of treatment groups, parasite species and isolate origin, and doses evaluating the efficacy of Credelio Quattro against ectoparasites

Study no.	No. dogs/group	Treatment group	Dose	Parasite	Isolate origin
1	10	Control	0 mg/kg	*C. felis*	USA
	10	Credelio Quattro	Lotilaner 20–30 mg/kg	*C. felis*	
			Moxidectin 0.02–0.03 mg/kg		
			Praziquantel 5.0–7.5 mg/kg		
			Pyrantel 5.0–7.5 mg/kg		
2	10	Control	0 mg/kg	*R. sanguineus*	USA
	10	Credelio Quattro	Lotilaner 20–30 mg/kg	*R. sanguineus*	
			Moxidectin 0.02–0.03 mg/kg		
			Praziquantel 5.0–7.5 mg/kg		
			Pyrantel 5.0–7.5 mg/kg		
	10	Lotilaner	20–30 mg/kg	*R. sanguineus*	
	10	Pyrantel	5.0–7.5 mg/kg	*R. sanguineus*	
3	10	Control	0 mg/kg	*R. sanguineus*	USA
	10	Credelio Quattro	Lotilaner 20–30 mg/kg	*R. sanguineus*	
			Moxidectin 0.02–0.03 mg/kg		
			Praziquantel 5.0–7.5 mg/kg		
			Pyrantel 5.0–7.5 mg/kg		
4	10	Control	0 mg/kg	*R. sanguineus*	Greece
	10	Credelio Quattro	Lotilaner 20–30 mg/kg	*R. sanguineus*	
			Moxidectin 0.02–0.03 mg/kg		
			Praziquantel 5.0–7.5 mg/kg		
			Pyrantel 5.0–7.5 mg/kg		

Treatments were administered orally on Day 0 to dogs in a fed state utilizing body weights collected on Day −5. Dogs were administered a single or a combination of tablets to achieve as close to the minimum target dosages of 20 mg/kg lotilaner, 0.02 mg/kg moxidectin, 5 mg/kg praziquantel, and 5 mg/kg pyrantel (Credelio Quattro), 20 mg/kg lotilaner alone (Study 2), or 5 mg/kg pyrantel alone (Study 2) without underdosing.

### Flea infestations and counts

To evaluate efficacy against pre-existing flea infestations and residual efficacy after treatment, 100 unfed adult *C. felis* (50% female/50% male) were experimentally infested on Days −1, 6, 13, 20, 29, and 35 (Study 1). Fleas utilized for experimental infestation were isolated from the field in 2004 and genetically enriched with wild-caught fleas within 3 years of study initiation. All fleas were removed from each dog by whole-body combing 24 h post-dose on Day 1 or 24 h post-infestation on Days 7, 14, 21, 30, and 36 according to standard operating procedures of the laboratory. After combing, fleas collected were characterized as live or dead, and enumerated and recorded for each dog. To prevent cross-contamination between treatment groups, freshly cleaned combs, disposable gloves, and aprons were used and changed after each dog.

### Tick infestations and counts

A total of three studies were conducted to evaluate the efficacy against pre-existing *R. sanguineus* infestations and residual efficacy after treatment. On Days −2, 5, 12, 19, and 30, each dog was experimentally infested with 50 unfed adult *R. sanguineus* (50% female/50% male). Ticks used for experimental infestation were last genetically enriched with wild-caught ticks within 5 years of Studies 2 and 4 and within 2 years of Study 3. All ticks were removed from each dog by whole-body combing 48 h post-dose on Day 2 or 48 h post-infestation on Days 7, 14, 21, and 32 according to standard operating procedures of the laboratory. After combing, ticks collected were characterized as dead or live, attached or unattached, and enumerated and recorded for each dog. To prevent cross-contamination between treatment groups, freshly cleaned combs, disposable gloves, and aprons were used and changed after each dog.

### Safety assessments

Physical examinations were performed and general health assessments conducted at least once daily prior to dose administration on Day 0 for all study animals. On the day of treatment, dogs were observed just prior to dose administration on Day 0 and at 1, 2, 4, and 8 h post-dosing. Starting on Day 1, general health assessments were conducted at least twice daily, continuing through the end of each study. Any abnormal observation after dosing, whether or not considered to be product-related, was recorded as an adverse event (AE) and followed until resolution, with any concomitant therapies administered recorded. Relationship to treatment was assessed based on the known safety profile of Credelio Quattro and timing of the event.

### Statistical analysis

All statistical analyses were performed using SAS version 9.4 software (SAS Institute, Cary, NC, USA), with the individual dog being the experimental unit. Hypotheses were tested at a two-sided 0.05 level of significance. Adequacy of infestation, assessed at each time point, was defined as ≥ 25% retention of live ticks or ≥ 50% retention of live fleas on at least six control dogs. Mean infestation rates in the control group were calculated as 100 × (total number of live ectoparasites recovered/total number infested). Efficacy for the treated groups at each time point was determined using the least-squares means [(LSM), marginal means] calculated from a linear mixed model. This model utilized live (attached and unattached) tick counts or live flea counts, respectively, as the response and treatment group as a fixed effect with block included as a random effect, compared to the control group. Percent efficacy was then calculated as 100 × (LSM_control_ − LSM_treated_)/(LSM_control_). A linear mixed model was also fitted to assess the acaricidal effect of Credelio Quattro across all tick studies, with treatment group as the fixed effect and block within study as the random effect.

## Results

Adequacy of infestation criteria was met for all studies at all time points evaluated. A breakdown of the number of dogs with adequate infestations and mean infestation rates for each study is provided in Table 2. In Study 1, Credelio Quattro demonstrated 100% efficacy with no live fleas recovered through day 36 after administration (Table 3). The difference in LSM mean live flea counts recovered from the Credelio Quattro-treated group was statistically significant (*P* < 0.0001) as compared to the non-treated control group. In Studies 2–4, LSM mean live tick counts for *R. sanguineus* in the Credelio Quattro-treated group ranged from 0 to 0.7, providing ≥ 97.1% efficacy through day 32 after administration (Table 4). Pyrantel only, administered in Study 2, did not provide sufficient efficacy against *R. sanguineus*, with LSM mean live tick counts ranging from 10.9 to 19.9 recovered. Lotilaner provided ≥ 99.6% efficacy at all time points, confirming that the acaricidal efficacy in Credelio Quattro is achieved by the inclusion of lotilaner (Table 4; Additional file 1: Tables S1–S4. Count data for each study). Differences in LSM live *R. sanguineus* counts in the Credelio Quattro-treated group as compared to non-treated controls were statistically significant (*P* < 0.0001) at all time points, while the pyrantel-only-treated group was not statistically significant (*P* ≥ 0.0601, Table 5) compared to the control.

**Table 2:** Adequacy of infestation in control groups at each infestation time point for all studies

Study no.	Parasite	Number of dogs with adequate infestation/total number of dogs in control group (mean infestation rate^a^)							
		Day 1	Day 2	Day 7	Day 14	Day 21	Day 30	Day 32	Day 36
1	*C. felis*	10/10 (89.8%)	–	10/10 (93.9%)	10/10 (90.1%)	10/10 (85.9%)	10/10 (79.7%)	n/a	10/10 (83.2%)
2	*R. sanguineus*	–	9/10 (40.8%)	8/10 (40.8%)	9/10 (42.4%)	8/10 (43.6%)	n/a	10/10 (48.8%)	n/a
3	*R. sanguineus*	–	10/10 (68.0%)	10/10 (63.6%)	10/10 (53.0%)	10/10 (47.2%)	n/a	10/10 (49.4%)	n/a
4	*R. sanguineus*	–	10/10 (60.6%)	10/10 (54.4%)	10/10 (69.4%)	9/10 (63.6%)	n/a	8/10 (53.4%)	n/a

^a^Infestation rate = (total number of ectoparasites recovered/total number infested) × 100

**Table 3:** Efficacy of Credelio Quattro against *C. felis* based on LSM total live flea counts

Study no	Treatment group	LSM live flea counts (% efficacy)					
		Day 1	Day 7	Day 14	Day 21	Day 30	Day 36
1	Control	89.8	93.9	90.1	85.9	79.7	83.2
	Credelio Quattro	0 (100)	0 (100)	0 (100)	0 (100)	0 (100)	0 (100)
	*P*-value (test statistic)	< 0.0001 (*t*_(9)_ = 37.96)	< 0.0001 (*t*_(9)_ = 38.30)	< 0.0001 (*t*_(9)_ = 32.52)	< 0.0001 (*t*_(9)_ = 24.65)	< 0.0001 (*t*_(9)_ = 19.88)	< 0.0001 (*t*_(9)_ = 16.78)

**Table 4:** Efficacy of Credelio Quattro and pyrantel or lotilaner alone against *R. sanguineus* based on LSM total live tick counts

Study no.	Treatment group	LSM live tick counts (% efficacy)				
		Day 2	Day 7	Day 14	Day 21	Day 32
2	Control	20.4	20.4	21.2	21.8	24.4
	Credelio Quattro	0.1 (99.5)	0.1 (99.5)	0.2 (99.1)	0.1 (99.5)	0.7 (97.1)
	Pyrantel	19.9 (2.5)	10.9 (46.6)	13.7 (35.4)	18.6 (14.7)	15.7 (35.7)
	Lotilaner	0 (100)	0 (100)	0 (100)	0 (100)	0.1 (99.6)
3	Control	34.0	31.8	26.5	23.6	24.7
	Credelio Quattro	0.1 (99.7)	0 (100)	0 (100)	0 (100)	0 (100)
4	Control	30.3	27.2	34.7	31.8	26.7
	Credelio Quattro	0 (100)	0 (100)	0.1 (99.7)	0 (100)	0 (100)

**Table 5:** Comparison of LSM mean total live *R. sanguineus* tick counts for all studies combined

Day	Control versus Credelio Quattro^a^		Control versus pyrantel^b^		Control versus lotilaner^b^	
	Efficacy (%)	*P*-value (test statistic)	Efficacy (%)	*P*-value (test statistic)	Efficacy (%)	*P*-value (test statistic)
2	99.8	< 0.0001 (*t*_(29)_ = 12.95)	2.5	0.8604 (*t*_(9)_ = 0.18)	100	0.0007 (*t*_(9)_ = 5.05)
7	99.9	< 0.0001 (*t*_(29)_ = 14.27)	46.6	0.0757 (*t*_(29)_ = 2.01)	100	< 0.0001 (*t*_(9)_ = 6.63)
14	99.6	< 0.0001 (*t*_(29)_ = 14.47)	35.4	0.0609 (*t*_(29)_ = 2.14)	100	< 0.0001 (*t*_(9)_ = 7.89)
21	99.9	< 0.0001 (*t*_(29)_ = 11.41)	14.7	0.2033 (*t*_(9)_ = 1.37)	100	0.0005 (*t*_(9)_ = 5.24)
32	99.1	< 0.0001 (*t*_(29)_ = 12.76)	35.7	0.0601 (*t*_(9)_ = 2.15)	99.6	0.0001 (*t*_(9)_ = 6.55)

^a^Data from Studies 2, 3, and 4 analyzed

^b^Data from Study 2 analyzed

AEs were reported in both the non-treated control and Credelio Quattro-treated groups. In the non-treated control group in Study 1, two dogs (20%) were reported with skin disorders, one with mild dermatitis in the right inguinal area requiring topical gentamicin treatment on Day 7, and one with alopecia with scales on the base of the tail on Day 35 not requiring treatment. These AEs were not associated with treatment administration. Lameness was reported in two Credelio Quattro-treated dogs (20%) 9 and 34 days post-treatment, which were treated with oral carprofen, and one dog was reported to have dermatitis in the right inguinal area not requiring treatment, 28 days post-treatment. These AEs were not likely related to treatment administration. Vomiting and bloody diarrhea were reported in one dog (10%) starting 3 days post-treatment with Credelio Quattro in Study 3 and was treated with oral metronidazole twice daily for 4 days, resolving thereafter. Despite timing suggestive of an alternative etiology, the gastrointestinal signs were categorized as possibly treatment-related, consistent with the known safety profile of Credelio Quattro. Lastly, one dog (10%) in Study 4 treated with Credelio Quattro was reported to have a wound on the chest which was treated with a topical antibiotic/steroid ointment and carprofen and which was not considered related to treatment.

## Discussion

The efficacy and safety of Credelio Quattro, a novel combination endectocide containing lotilaner, moxidectin, praziquantel, and pyrantel, was evaluated against *C. felis* and the least susceptible tick species for lotilaner, *R. sanguineus*, in laboratory studies. The results indicate that Credelio Quattro is highly effective for the treatment and control of *C. felis,* providing 100% efficacy at all time points evaluated through Day 36. The robust pulicidal activity of lotilaner contained in Credelio Quattro and the monoproduct Credelio is complemented by a rapid onset of efficacy after administration for existing infestations and persistent rapid speed of kill throughout the entire month. Previous studies have demonstrated onset of efficacy of 64.0%, 89.9%, and 99.2% against pre-existing *C. felis* infestations at 2, 4, and 6 h after administration with 20 mg/kg oral lotilaner in dogs, providing timely efficacy and relief to dogs at the time of administration [29, 30]. In addition, lotilaner demonstrated persistent speed of kill, with 97.1% efficacy 4 h after *C. felis* infestation on Day 35, thereby continuing to provide rapid elimination of infestation for the entire month [30].

The efficacy of lotilaner as the active ingredient against ectoparasites and non-interference of the additional active ingredients contained in Credelio Quattro, moxidectin, praziquantel and pyrantel, were demonstrated by the *R. sanguineus* efficacy results for Credelio Quattro and lotilaner alone in Study 2. The inclusion of pyrantel in this non-interference study was sufficient, as previous studies had confirmed the inactivity of praziquantel in vitro and the insufficient efficacy of moxidectin in vivo against both *C. felis* and *R. sanguineus* [26].

Lotilaner in Credelio Quattro provides established activity against fleas and ticks and is included at the same dose as in the monoproduct Credelio, preserving its rapid and persistent ectoparasiticidal performance. Moxidectin and pyrantel broaden the spectrum to heartworm and gastrointestinal nematodes, with Credelio Quattro demonstrating high efficacy against hookworm and roundworm infections [31–33] and being labeled for the prevention of heartworm disease caused by *Dirofilaria immitis*. Praziquantel adds cestocidal activity, providing effective treatment of tapeworm infections, including *D. caninum*, *Taenia pisiformis*, and *Echinococcus* spp. [34]. Thus, the four active ingredients in Credelio Quattro complement each other to deliver broad-spectrum endectocidal coverage against fleas, ticks, heartworm, gastrointestinal nematodes, and tapeworms in a single monthly oral product, and the non-interference studies confirm that co-formulation does not compromise the efficacy or speed of kill of the individual components [31, 32, 34].

Against the least susceptible tick species for lotilaner, *R. sanguineus*, Credelio Quattro provided ≥ 97.1% efficacy for the entire month. As Credelio Quattro contains the same dose of lotilaner as the monoproduct Credelio, this confirms that the exposure of lotilaner in this combination product also provides robust efficacy against more susceptible tick species *I. scapularis*, *D. variabilis*, and *A. americanum*, which Credelio is currently labeled against [35]. Additionally, the speed of kill of lotilaner contained in Credelio Quattro and Credelio against *I. scapularis* provided protection against the transmission of *Borrelia burgdorferi* in dogs [36]. No dogs treated with Credelio tested positive for *B. burgdorferi* on SNAP 4Dx Plus, Lyme Quant C_6_^®^ test (IDEXX Laboratories, Inc., Westbrook, ME, USA) or polymerase chain reaction (PCR) analysis of skin biopsies, while only one dog in the Credelio Quattro-treated group tested positive for *B. burgdorferi* on SNAP 4Dx Plus at one time point and negative on all other tests after infestation with wild-caught *I. scapularis* experimentally infested 28 days after treatment [36].

The AEs reported in both the control and treatment groups, including dermatitis and gastrointestinal symptoms, align with previously documented AEs in both field studies and a laboratory target animal safety study performed in 8-week-old puppies [37]. These findings suggest that Credelio Quattro is well tolerated in the canine population, providing a safe and efficacious endectocide option for pet owners to provide the broadest protection against parasites.

The efficacy of Credelio Quattro against *C. felis* (100%) and *R. sanguineus* (≥ 97.0%) in these laboratory studies is comparable to that of other commercially available endectocides. For instance, Simparica Trio^®^ (sarolaner, moxidectin, pyrantel, Zoetis, Kalamazoo, MI, USA) demonstrated flea efficacy of ≥ 99.7% throughout the month and efficacy against *R. sanguineus* of ≥ 94.0% in the USA and ≥ 97.4% in Europe [38, 39]. While direct comparisons are complex, the isoxazoline class as a whole shows robust ectoparasiticidal activity. Notably, lotilaner demonstrates a key advantage in its persistent speed of kill against certain tick species. Against *A. americanum* infestations 28 days after treatment, lotilaner achieved 92.3% efficacy by 24 h post-infestation, whereas sarolaner (Simparica Trio^®^) and afoxolaner (NexGard^®^, Boehringer Ingelheim, Duluth, GA, USA) achieved 4.9% and 0.0%, respectively, over the same period [40]. Furthermore, the ability to prevent the transmission of *B. burgdorferi* from infected *I. scapularis* ticks to dogs is demonstrated across the isoxazoline class, including Credelio Quattro and Credelio [36], NexGard^®^ Plus [41], and Simparica^®^ (Zoetis, Kalamazoo, MI, USA) [42].

Certain limitations of the present studies should be considered. The evaluations were conducted under controlled laboratory conditions using established flea and tick isolates, which, while ensuring consistency and minimizing external variables, may not fully replicate the diverse environmental challenges and infestation pressures encountered by dogs in a real-world field setting. However, the efficacy of lotilaner under such conditions has been confirmed in previous field studies [43–45]. While laboratory studies of a 1-month duration are a standard and necessary approach for regulatory approval, these referenced field studies also confirm the effectiveness of lotilaner with longer-term, consecutive use. Likewise, while pathogen transmission was not studied in the laboratory studies presented here, the ability of lotilaner to prevent transmission of *Babesia canis* from infected *D. reticulatus* ticks has been demonstrated previously [46], with additional, recent studies [36] demonstrating both Credelio and Credelio Quattro preventing transmission of *B. burgdorferi* from wild-caught infected *I. scapularis* ticks.

## Conclusions

These laboratory studies confirm the safety and effectiveness of a single dose of Credelio Quattro, a novel oral combination chewable tablet, at the minimum effective dosages of 20 mg/kg lotilaner, 0.02 mg/kg moxidectin, 5 mg/kg praziquantel, and pyrantel for the treatment and control of *C. felis* and the dose-limiting tick species, *R. sanguineus,* on dogs for 1 month.

## Supplementary Information

Additional file 1: Tables S1–S4. Parasite count for each dog at each time point, by study.

## Data Availability

Data supporting the conclusions of this article are included within the article.

## Abbreviations

AE: Adverse event
CAPC: Companion Animal Parasite Council
ESCCAP: European Scientific Counsel Companion Animal Parasites
LSM: Least-squares mean
WAAVP: World Association for the Advancement of Parasitology
