# Supplementary material for: Efficacy of Credelio Quattro™ chewable tablets containing lotilaner, moxidectin, pyrantel, and praziquantel against Ctenocephalides felis and Rhipicephalus sanguineus infestations on dogs
Source: Parasit Vectors. 2026 Apr 27;19:246. doi: 10.1186/s13071-026-07405-1 (PMC13255492; doi:10.1186/s13071-026-07405-1)
Supplement: Supplementary file 1 — Additional file 1: Tables S1–S4. Parasite count for each dog at each time point, by study. [file 13071_2026_7405_MOESM1_ESM.docx]

**Supplemental Information: Raw data counts of fleas and ticks, by study.**

*Table S1. Ctenocephalides felis Counts (Study 1)*

| Control Group | | | | | | | |
| --- | --- | --- | --- | --- | --- | --- | --- |
| Day | | | | | | | |
| Dog | -5 | 1 | 7 | 14 | 21 | 30 | 36 |
| 1 | 78 | 100 | 99 | 97 | 97 | 96 | 96 |
| 2 | 82 | 96 | 93 | 100 | 86 | 91 | 90 |
| 3 | 63 | 96 | 100 | 79 | 100 | 84 | 103 |
| 4 | 67 | 100 | 100 | 95 | 79 | 85 | 78 |
| 5 | 91 | 79 | 91 | 91 | 83 | 56 | 60 |
| 6 | 69 | 89 | 87 | 98 | 90 | 67 | 60 |
| 7 | 68 | 82 | 77 | 75 | 67 | 76 | 70 |
| 8 | 75 | 90 | 89 | 90 | 96 | 94 | 100 |
| 9 | 77 | 81 | 102 | 95 | 71 | 74 | 90 |
| 10 | 87 | 90 | 98 | 81 | 90 | 74 | 85 |
| Mean | 75.7 | 89.8 | 93.9 | 90.1 | 85.9 | 79.7 | 83.2 |
| GeoMean | 75.2 | 89.5 | 93.6 | 89.7 | 85.2 | 78.7 | 81.8 |
| Treatment Group | | | | | | | |
| Day | | | | | | | |
| Dog | -5 | 1 | 7 | 14 | 21 | 30 | 36 |
| 1 | 69 | 0 | 0 | 0 | 0 | 0 | 0 |
| 2 | 80 | 0 | 0 | 0 | 0 | 0 | 0 |
| 3 | 78 | 0 | 0 | 0 | 0 | 0 | 0 |
| 4 | 80 | 0 | 0 | 0 | 0 | 0 | 0 |
| 5 | 90 | 0 | 0 | 0 | 0 | 0 | 0 |
| 6 | 88 | 0 | 0 | 0 | 0 | 0 | 0 |
| 7 | 65 | 0 | 0 | 0 | 0 | 0 | 0 |
| 8 | 72 | 0 | 0 | 0 | 0 | 0 | 0 |
| 9 | 68 | 0 | 0 | 0 | 0 | 0 | 0 |
| 10 | 66 | 0 | 0 | 0 | 0 | 0 | 0 |
| Mean | 75.6 | 0 | 0 | 0 | 0 | 0 | 0 |
| GeoMean | 75.1 | 0 | 0 | 0 | 0 | 0 | 0 |

*Table S2. Rhipicephalus sanguineus (US) Total Live Tick Counts (Study 2)*

| Control Group | | | | | | |
| --- | --- | --- | --- | --- | --- | --- |
| Day | | | | | | |
| Dog | -5 | 2 | 7 | 14 | 21 | 32 |
| 1 | 19 | 21 | 21 | 21 | 34 | 22 |
| 2 | 16 | 13 | 14 | 26 | 14 | 24 |
| 3 | 14 | 27 | 17 | 16 | 30 | 14 |
| 4 | 17 | 20 | 30 | 22 | 29 | 42 |
| 5 | 24 | 14 | 1 | 13 | 10 | 12 |
| 6 | 28 | 50 | 30 | 34 | 49 | 44 |
| 7 | 17 | 24 | 23 | 26 | 18 | 34 |
| 8 | 17 | 19 | 10 | 31 | 13 | 14 |
| 9 | 31 | 16 | 29 | 6 | 9 | 22 |
| 10 | 14 | 0 | 29 | 17 | 12 | 16 |
| Mean | 19.7 | 20.4 | 20.4 | 21.2 | 21.8 | 24.4 |
| GeoMean | 19.0 | 15.1 | 16.5 | 19.4 | 18.8 | 22.2 |
| Credelio Quattro Treatment Group | | | | | | |
| Day | | | | | | |
| Dog | -5 | 2 | 7 | 14 | 21 | 32 |
| 1 | 20 | 0 | 0 | 1 | 0 | 0 |
| 2 | 21 | 0 | 0 | 0 | 0 | 0 |
| 3 | 15 | 0 | 0 | 0 | 0 | 0 |
| 4 | 17 | 0 | 0 | 0 | 0 | 3 |
| 5 | 15 | 0 | 0 | 0 | 0 | 0 |
| 6 | 19 | 1 | 1 | 0 | 0 | 3 |
| 7 | 16 | 0 | 0 | 0 | 0 | 0 |
| 8 | 24 | 0 | 0 | 1 | 0 | 0 |
| 9 | 13 | 0 | 0 | 0 | 1 | 1 |
| 10 | 32 | 0 | 0 | 0 | 0 | 0 |
| Mean | 19.2 | 0.1 | 0.1 | 0.2 | 0.1 | 0.7 |
| GeoMean | 18.6 | 0.1 | 0.1 | 0.1 | 0.1 | 0.4 |
| Lotilaner only Treatment Group | | | | | | |
| Day | | | | | | |
| Dog | -5 | 2 | 7 | 14 | 21 | 32 |
| 1 | 17 | 0 | 0 | 0 | 0 | 0 |
| 2 | 14 | 0 | 0 | 0 | 0 | 0 |
| 3 | 19 | 0 | 0 | 0 | 0 | 1 |
| 4 | 23 | 0 | 0 | 0 | 0 | 0 |
| 5 | 15 | 0 | 0 | 0 | 0 | 0 |
| 6 | 36 | 0 | 0 | 0 | 0 | 0 |
| 7 | 26 | 0 | 0 | 0 | 0 | 0 |
| 8 | 16 | 0 | 0 | 0 | 0 | 0 |
| 9 | 18 | 0 | 0 | 0 | 0 | 0 |
| 10 | 14 | 0 | 0 | 0 | 0 | 0 |
| Mean | 19.8 | 0.0 | 0.0 | 0.0 | 0.0 | 0.1 |
| GeoMean | 19.0 | 0.0 | 0.0 | 0.0 | 0.0 | 0.1 |
| Pyrantel only Treatment Group | | | | | | |
| Day | | | | | | |
| Dog | -5 | 2 | 7 | 14 | 21 | 32 |
| 1 | 15 | 29 | 24 | 26 | 34 | 16 |
| 2 | 16 | 21 | 3 | 14 | 13 | 10 |
| 3 | 17 | 13 | 7 | 17 | 17 | 2 |
| 4 | 19 | 6 | 1 | 0 | 14 | 26 |
| 5 | 23 | 16 | 18 | 16 | 11 | 7 |
| 6 | 25 | 49 | 34 | 26 | 48 | 30 |
| 7 | 18 | 15 | 6 | 3 | 13 | 17 |
| 8 | 32 | 15 | 3 | 0 | 7 | 15 |
| 9 | 16 | 18 | 13 | 18 | 14 | 28 |
| 10 | 13 | 17 | 0 | 17 | 15 | 6 |
| Mean | 19.4 | 19.9 | 10.9 | 13.7 | 18.6 | 15.7 |
| GeoMean | 18.8 | 17.5 | 6.3 | 8.2 | 16.1 | 12.6 |

*Table S3. R. sanguineus (US) Total Live Tick Counts (Study 3)*

| Control Group | | | | | | |
| --- | --- | --- | --- | --- | --- | --- |
| Day | | | | | | |
| Dog | -5 | 2 | 7 | 14 | 21 | 32 |
| 1 | 28 | 35 | 45 | 35 | 24 | 27 |
| 2 | 27 | 34 | 24 | 31 | 20 | 24 |
| 3 | 42 | 37 | 36 | 22 | 30 | 22 |
| 4 | 32 | 29 | 26 | 22 | 199 | 24 |
| 5 | 25 | 51 | 22 | 24 | 20 | 18 |
| 6 | 25 | 31 | 37 | 29 | 22 | 31 |
| 7 | 15 | 26 | 23 | 23 | 16 | 26 |
| 8 | 21 | 39 | 33 | 23 | 30 | 21 |
| 9 | 38 | 32 | 43 | 32 | 32 | 33 |
| 10 | 35 | 26 | 29 | 24 | 23 | 21 |
| Mean | 28.8 | 34.0 | 31.8 | 26.5 | 23.6 | 24.7 |
| GeoMean | 27.7 | 33.4 | 30.9 | 26.1 | 23.1 | 24.3 |
| Credelio Quattro Treatment Group | | | | | | |
| Day | | | | | | |
| Dog | -5 | 2 | 7 | 14 | 21 | 32 |
| 1 | 27 | 0 | 0 | 0 | 0 | 0 |
| 2 | 34 | 0 | 0 | 0 | 0 | 0 |
| 3 | 22 | 0 | 0 | 0 | 0 | 0 |
| 4 | 22 | 0 | 0 | 0 | 0 | 0 |
| 5 | 35 | 0 | 0 | 0 | 0 | 0 |
| 6 | 21 | 0 | 0 | 0 | 0 | 0 |
| 7 | 31 | 0 | 0 | 0 | 0 | 0 |
| 8 | 29 | 0 | 0 | 0 | 0 | 0 |
| 9 | 25 | 1 | 0 | 0 | 0 | 0 |
| 10 | 50 | 0 | 0 | 0 | 0 | 0 |
| Mean | 29.6 | 0.1 | 0.0 | 0.0 | 0.0 | 0.0 |
| GeoMean | 28.6 | 0.1 | 0.0 | 0.0 | 0.0 | 0.0 |

*Table S4. R. sanguineus (EU) Total Live Tick Counts (Study 4)*

| Control Group | | | | | | |
| --- | --- | --- | --- | --- | --- | --- |
| Day | | | | | | |
| Dog | -5 | 2 | 7 | 14 | 21 | 32 |
| 1 | 50 | 17 | 29 | 38 | 47 | 38 |
| 2 | 28 | 13 | 26 | 20 | 19 | 20 |
| 3 | 24 | 21 | 14 | 14 | 14 | 3 |
| 4 | 47 | 33 | 18 | 41 | 36 | 43 |
| 5 | 33 | 22 | 20 | 28 | 4 | 9 |
| 6 | 44 | 41 | 40 | 42 | 42 | 41 |
| 7 | 38 | 31 | 35 | 39 | 36 | 33 |
| 8 | 42 | 40 | 41 | 50 | 50 | 38 |
| 9 | 32 | 39 | 31 | 49 | 40 | 29 |
| 10 | 36 | 46 | 18 | 26 | 30 | 13 |
| Mean | 37.4 | 30.3 | 27.2 | 34.7 | 31.8 | 26.7 |
| GeoMean | 36.5 | 28.2 | 25.7 | 32.5 | 26.8 | 21.5 |
| Credelio Quattro Treatment Group | | | | | | |
| Day | | | | | | |
| Dog | -5 | 2 | 7 | 14 | 21 | 32 |
| 1 | 36 | 0 | 0 | 0 | 0 | 0 |
| 2 | 46 | 0 | 0 | 0 | 0 | 0 |
| 3 | 52 | 0 | 0 | 0 | 0 | 0 |
| 4 | 42 | 0 | 0 | 0 | 0 | 0 |
| 5 | 39 | 0 | 0 | 0 | 0 | 0 |
| 6 | 22 | 0 | 0 | 1 | 0 | 0 |
| 7 | 27 | 0 | 0 | 0 | 0 | 0 |
| 8 | 36 | 0 | 0 | 0 | 0 | 0 |
| 9 | 29 | 0 | 0 | 0 | 0 | 0 |
| 10 | 49 | 0 | 0 | 0 | 0 | 0 |
| Mean | 37.8 | 0.0 | 0.0 | 0.1 | 0.0 | 0.0 |
| GeoMean | 36.6 | 0.0 | 0.0 | 0.1 | 0.0 | 0.0 |
